# Palliative radiotherapy

**DOI:** 10.1136/bmj.k821

**Published:** 2018-03-23

**Authors:** Katie Spencer, Rhona Parrish, Rachael Barton, Ann Henry

**Affiliations:** 1 Leeds Institute of Cancer and Pathology, University of Leeds, Leeds LS2 9NL, UK; 2 Garforth Medical Centre, Garforth, Leeds LS25 1HB, UK; 3 Queen’s Centre for Oncology and Haematology, Castle Hill Hospital, Cottingham HU16 5JQ, UK

**What you need to know**

- Palliative radiotherapy offers effective symptom control for focal disease due to cancer
- Increased analgesia, anti-emetics, and in some cases corticosteroids can help to reduce discomfort and side effects
- Acute side effects of radiotherapy usually resolve within 4-6 weeks of completing treatment
- Symptoms of cancer may deteriorate before improvement
- For patients in the final weeks of life, the side effects and disruption of palliative radiotherapy may outweigh the benefits, and holistic palliative care may be more appropriate

Palliative radiotherapy offers a quick, inexpensive, and effective way of reducing many of the focal symptoms of advanced, incurable cancer, whether these arise from the primary tumour or from metastatic deposits. It can improve quality of life while being associated with limited treatment burden in terms of both hospital attendances and side effects.^1^ The average UK general practice oversees care for around 20 patients with terminal cancer each year with higher numbers seen in secondary care,^2^ ^3^ while a Canadian survey of general practitioners found that 85% had provided care for patients with advanced cancer within the previous month.^4^ This article aims to update non-specialists on the benefits, practicalities, and side effects of palliative radiotherapy to ensure that patients are considered and referred for these treatments when appropriate.

**Sources and selection criteria**

In developing this article, we used multiple sources. For each of the sites treated, we carried out a search of the Cochrane database to identify systematic reviews. Search terms used included “palliative AND radiotherapy AND bone metastases,” “spinal cord compression AND radiotherapy,” and “palliative radiotherapy AND lung cancer.” Where no Cochrane reviews were identified, we used Medline searches to identify other relevant systematic reviews and individual studies. We also searched our existing collections of relevant references and consulted appropriate experts where relevant studies could not be identified. In all cases we used the highest level of evidence available to inform this review, with more recent studies cited where possible. All searches were carried out between September 2017 and January 2018.

## How is radiotherapy delivered?

**Fig 1:**
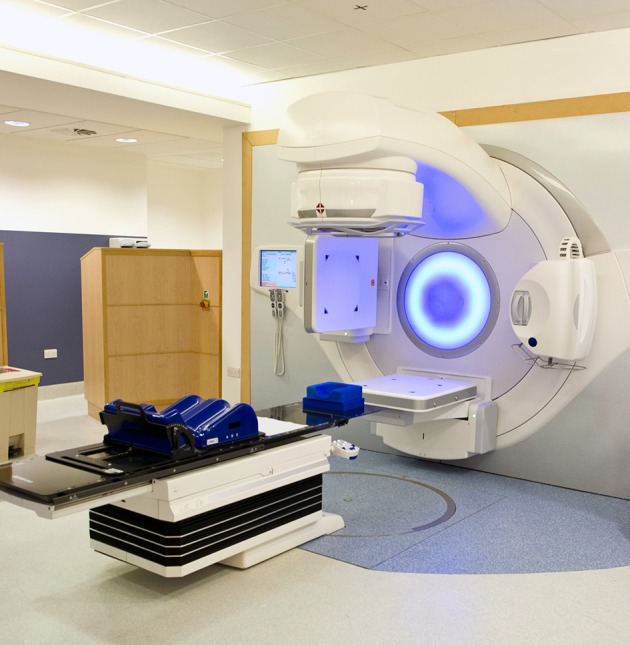
Linear accelerator used to deliver radiotherapy

Radiotherapy is delivered with linear accelerators (fig 1) in specialised cancer centres generally located in large urban areas (see box 1). High energy x rays are targeted to the disease site, causing DNA damage and cell death. Curative radiotherapy is routinely delivered over multiple, small daily doses (fractions) to reduce the risk of long term, permanent side effects in adjacent normal tissues.^5^ Palliative treatments require lower total doses, with the focus shifting to symptom control while minimising treatment burden. This change underpins the routine delivery of palliative radiotherapy using much shorter courses of larger fraction size (hypo-fractionation).

Box 1 **Practicalities of palliative radiotherapy**

**Fig 2:**
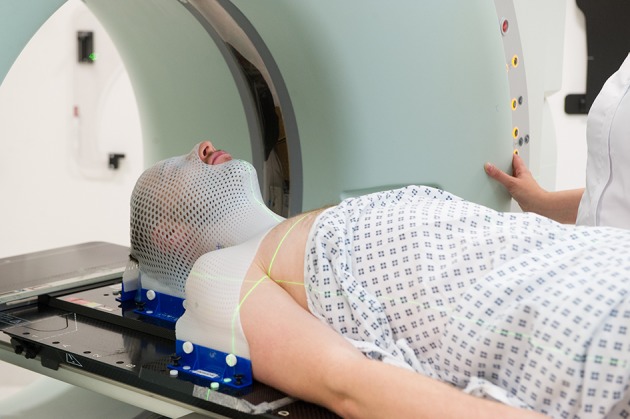
For radiotherapy to the head, neck, or upper chest, a close fitting mask maybe needed to ensure a consistent treatment position

- Anatomically targeted treatment during which the patient lies still on a relatively hard-topped treatment couch for about 15 minutes. The procedure itself is not associated with pain, but some may find the treatment position uncomfortable. Increased pain relief ahead of treatment can help. Occasionally this discomfort outweighs the benefits
- Patients must be able to provide informed consent. In emergency situations (such as spinal cord compression) a decision may be made in the patient’s best interests if the patient lacks capacity and has no available representative
- Patients must be able to follow verbal commands from radiographers outside the treatment room; a lack of capacity may make it difficult or even unsafe to deliver treatment. Sedation and anaesthesia are not routinely used for palliative radiotherapy
- Palliative treatments are usually delivered as a single dose or a short course (usually over 1-3 weeks)
- A close fitting mask maybe needed to ensure a consistent treatment position for treatments to the head, neck or upper chest (fig 2). This is generally well tolerated, even by more anxious patients
- Re-treatment may be possible for recurrent symptoms, but side effects may be greater
- Referrals and management of treatment related side effects can be discussed with the local radiotherapy department

**Fig 3:**
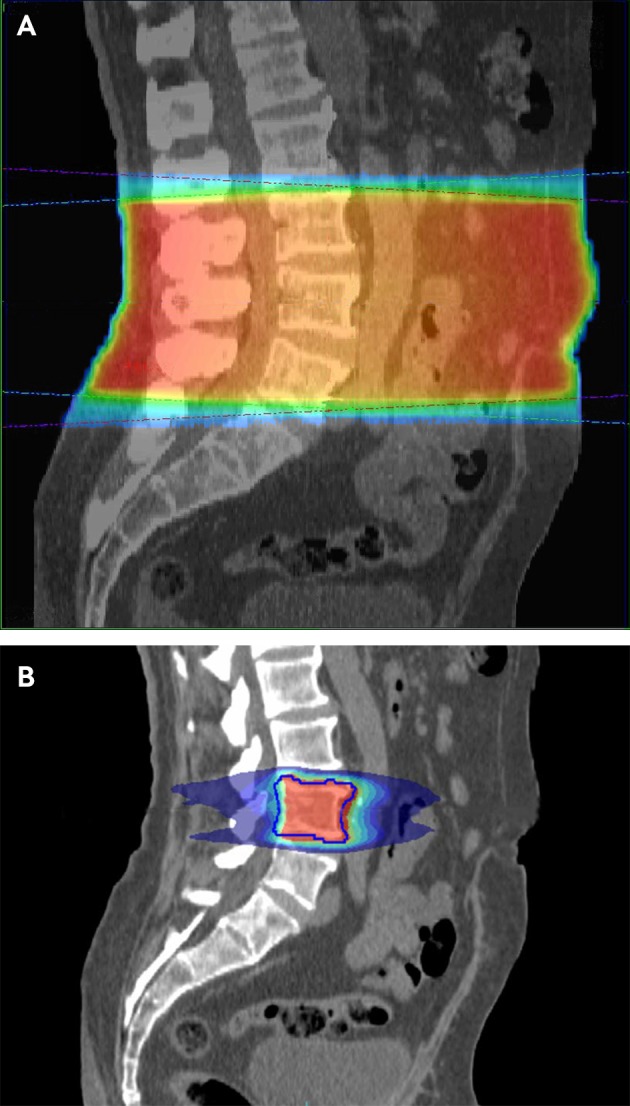
Computed tomograms showing the difference in radiotherapy dose distribution between simple, conventional palliative radiotherapy (A), and targeted stereotactic radiotherapy (B). The latter treatment plan allows a dose of roughly three times greater biological effectiveness to the target with significantly lower dose to surrounding tissue

Increasingly, advanced techniques are used to offer more precise treatment delivery, allowing increased dose to the tumour while maintaining limited dose to surrounding tissues (stereotactic radiotherapy) (see fig 3).

## What are the main barriers to referral for palliative radiotherapy?

Despite increasing numbers of radiotherapy treatment machines in the UK,^6^ national data highlight that radiotherapy use is lower than in Europe.^7^ ^8^ ^9^ ^10^ Internationally, multiple population based studies have shown that the chances of receiving palliative radiotherapy are dictated not only by clinical need but also by factors such as age, deprivation, and distance from treatment centre.^11^ ^12^ Questionnaire based studies suggest that a lack of understanding of the benefits of palliative radiotherapy among general practitioners and palliative care specialists may also be a barrier to referral.^13^ ^14^ ^15^

## What are the indications for using palliative radiotherapy?

A wide range of focal symptoms from advanced cancer can be treated with palliative radiotherapy as described below (and in table 1). Patients can undergo radiotherapy alongside palliative systemic anticancer treatments.

**Table 1:** Benefits of palliative radiotherapy for varying indications (evidence referenced is the highest level identified)

Treatments assessed	Study and sample size	Endpoints	Results
**Pain due to bone metastases**			
Single fraction radiotherapy *v* longer, more fractionated courses.	Chow et al 2012^16^ (SR, 5617 patients, 25 trials)	Pain response, re-treatment rate, pathological fracture rate. Time point varied between trials	60.7% response rate (OR for single *v* multiple fraction treatments 0.98 (95% CI 0.95 to 1.02)). 23.8% complete pain resolution. Re-treatment higher after single fraction (OR 2.6 (1.92 to 3.47)) No significant difference in pathological fracture rate (overall 3.2%, OR 1.10 (0.65 to 1.86))
	Sze et al 2004^17^ (SR, 3487 painful sites, 11 trials)	Pain response, re-treatment rate, pathological fracture rate. Time point varied between trials	59% response rate (OR for single *v* multiple fractions 1.03 (0.89 to 1.19)). 33% complete pain resolution. Re-treatment rate higher after single fraction (21.5% *v* 7.4%, OR 3.44 (2.67 to 4.43)). Fracture rate higher after single fraction (3% *v* 1.6%, OR 1.82 (1.06 to 3.11))
	Steenland et al 1999^18^ (RCT, 1157 patients)	Pain response in remaining lifespan (1° endpoint), re-treatment rate, pathological fracture rate. Assessed weekly	71% response rate; 35% complete resolution of pain; median time to benefit 3 weeks. Re-treatment rate higher after single fraction (25% *v* 7% (P<0.0001)). Fracture rate higher after single fraction (4% *v* 2% (P<0.05))
Single 8 Gy fraction of radiotherapy *v* ibandronate infusion in metastatic prostate cancer	Hoskin et al 2015^19^ (RCT, 470 patients)	Pain response at 4 weeks (1° endpoint), crossover, pathological fracture rate. Assessed 4 weekly	53.1% response with radiotherapy *v* 49.5% with ibandronate (difference 3.7% (−12.4% to 5.0%), P=0.49). 24% crossover with ibandronate *v* 31% with radiotherapy. 3% fracture rate with ibandronate *v* 2% with radiotherapy (P=0.31)
**Locally advanced lung cancer**			
Various palliative radiotherapy regimens	Stevens et al 2015^20^ (SR, 3576 patients, 14 RCTs)	Control of thoracic symptoms, overall survival Time point varied between trials	Pooled symptom response rates not reported due to study heterogeneity. Possibly better 1 year overall survival with higher dose regimens for patients with good performance status (33.3% (11.4% to 46.2%) *v* 25.6% (9.4% to 45.7%)), but unclear due to high study heterogeneity (n=1081, 8 trials). No survival improvement seen in poor performance status patients (risk ratio 0.96 (0.91 to 1.02) (n=911, 7 trials)
Various palliative radiotherapy regimens	Fairchild et al 2008^21^ (SR, 3473 patients, 13 RCTs)	Control of thoracic symptoms, overall survival Time point varied between trials	After high and low dose regimens, complete resolution of haemoptysis reported by 73.7% *v* 68.9% (P=0.19), improvement reported by 80.2% *v* 81.2%; 48.2% *v* 53.5% reported improved cough (P=0.04); 57.5% *v* 51.9% improved chest pain (P=0.43 with significant heterogeneity between studies). Individual RCTs reported improvement in shortness of breath in 35-40%^22^-^24^ One trial reported median time to response 5-7 weeks.^24^ 1 year overall survival higher with high dose regimens (26.5% *v* 21.7%, P=0.002), at the expense of significantly increased oesophagitis
**Locally advanced oesophageal and gastric cancer**			
External radiotherapy (40 Gy in 20 fractions, twice daily)	Kassam et al 2008^25^ (phase I/II, 39 patients)	Dysphagia response at 56 days, survival, toxicity	Improved swallowing function reported by 69%, median time to benefit 4 weeks, duration of response 5.5 months
Oesophageal stenting with or without external radiotherapy	Javed et al 2010^26^ (RCT, 84 patients)	Duration of dysphagia relief after stenting, overall survival	Duration of dysphagia relief increased with radiotherapy (7 *v* 3 months, P=0.002). Median overall survival increased with radiotherapy (180 *v* 120 days, P=0.009)
Oesophageal brachytherapy with or without external radiotherapy	Rosenblatt et al 2010^27^ (RCT, 219 patients)	Dysphagia relief, overall survival	Improved duration of dysphagia relief with radiotherapy: at 200 days, 69.6% had not experienced a dysphagia event *v* 51.8% without radiotherapy (P=0.014 in multivariable modelling). No significant improvement in overall survival
Palliative radiotherapy for advanced gastric cancer	Tey et al 2017^28^ (SR, 122 patients, 7 retrospective studies)	Reduction in gastric bleeding (response definitions varied)	Gastric bleeding reduced in 74% of patients (pooled analysis). Small numbers reported for pain and obstruction responses (n=18 and 33)
**Malignant spinal cord compression**			
20 Gy in 5 fractions *v* 30 Gy in 10 fractions radiotherapy	Rades et al 2016^29^ (RCT, 203 patients (155 assessable))	Motor function at 1 month, local control, overall survival	No significant differences in mobility (P=0.86), local control (P=0.51), or survival (P=0.68). 41.3% reported improved motor function after treatment, and 47.1% remained stable. Improvement in ambulation not reported. Median overall survival 3.2 months
8 Gy single fraction *v* 16 Gy in 2 fractions radiotherapy	Maranzano et al 2009^30^ (RCT, 327 patients (303 assessable))	Symptom control (pain, motor, and sphincter function) at 1 month, toxicity, duration of response, overall survival	No significant difference in response rates or duration (P=0.40): median duration of response 5 months; median overall survival 4 months. 53% (95% CI 47% to 58%) achieved a pain response (25% (21% to 31%) complete resolution). 27% of non-ambulatory patients regained mobility after treatment (only 4% for those with paraplegia before treatment). 27% with sphincter disturbance regained control. Acute side effects were equivalent
16 Gy in 2 fractions *v* split course (total dose 30 Gy in 8 fractions)	Maranzano et al 2005^31^ (RCT, 300 patients (276 assessable))	Symptom control (pain, motor, and sphincter function) at 1 month, toxicity, duration of response, overall survival	No significant difference in response rates or duration: median duration of response 3.5 months; median overall survival 4 months. 56.9% (51.1% to 62.7%) achieved a pain response (33.3% (27.7% to 38.9%) complete resolution). 35% of non-ambulatory patients regained mobility (not anyone with paraplegia). 14% with sphincter disturbance regained control. Acute side effects were equivalent
Radiotherapy (30 Gy in 10 fractions) with or without surgical decompression	Patchell et al 2005^32^ (RCT, 101 patients (study stopped at interim analysis))	Mobility (time point unclear), continence, corticosteroid use, pain control, overall survival	Post-treatment ambulation rates 84% with surgery *v* 57% with radiotherapy alone (odds ratio 6.2 (2.0 to 19.8), P=0.001). Continence was more likely after surgery, and doses of corticosteroids (P=0.009) and opiates (P=0.002) were lower. Median survival 126 days after surgery *v* 100 days after radiotherapy alone (multivariable analysis HR 0.60 (0.38 to 0.96), P=0.033)
**Brain metastases**			
Whole brain radiotherapy (WBRT) (20 Gy in 5 fractions) *v* dexamethasone alone in non-small cell lung cancer	Mulvenna et al 2016^33^ (RCT, 538 patients)	Overall survival, quality of life (measured in QALYs), use of corticosteroids	All patients received dexamethasone. No difference in overall survival with or without WBRT (median survival 9.2 weeks *v* 8.5 weeks, HR 1.06 (0.90 to 1.26)), or quality of life (mean QALY 46.4 *v* 41.7 days).
Effectiveness and adverse events after WBRT for adults with multiple brain metastases	Tsao et al 2012^34^ (SR, 10 835 patients, 39 trials)	Overall survival, cerebral disease control, quality of life and symptom control	Unable to recommend one WBRT regimen over others due to lack of quality of life outcomes and no overall improvement in overall survival (n=3645, 8 trials). No improvement in survival (HR 1.08 (0.98 to 1.18)) or symptom control with addition of radio-sensitising drugs to WBRT. Toxicity increased. (n=2016, 6 trials). Addition of stereotactic radiotherapy to WBRT improved cerebral control (n=464, 3 trials). Improvement in overall survival only in individuals with a single metastasis and good performance status in one trial (n=333) (6.5 months *v* 4.9 months, P=0.03). Significantly reduced steroid doses after stereotactic radiotherapy shown in one trial (n=333) (52% *v* 33%, P=0.016). Addition of WBRT to stereotactic radiotherapy improved cerebral control (HR 2.61 (1.68 to 4.06), P<0.001) in pooled analysis (n=577, 3 trials) but not overall survival (HR 0.98 (0.71 to 1.35), P=0.88) (n=218, 2 trials)
Neurocognitive outcomes after stereotactic radiotherapy with or without WBRT.	Chang et al 2009^35^ (RCT, 58 patients (trial stopped early after interim analysis))	Neurocognitive outcomes at 4 months, cerebral disease control	Addition of WBRT resulted in lower CNS recurrence at 1 year (73% recurrence-free *v* 27%, P<0.001). This was at the cost of a higher probability of significantly reduced total recall at 4 months (mean posterior probability 52% *v* 24%). This difference persisted at 6 months. In practise, to avoid cognitive decline, regular MRI surveillance is often preferred over WBRT^36^
**Head and neck cancer**			
30 Gy in 5 fractions radiotherapy delivered every 3 days	Porceddu et al 2007^37^ (phase II, 37 patients)	Response rate, symptom control, quality of life, and toxicity. Overall and progression-free survival	80% had an objective response at 2 weeks after treatment, 67% reported improved pain control, 33% felt their ability to eat solids was improved, 62% reported improved overall quality of life. 74% of patients experienced significant dysphagia during treatment, resolving by 4 weeks later. Median overall survival was 6.1 months (range 0.5–21) and progression-free survival 3.9 months (0.5–21).
42 Gy in 12 fractions radiotherapy delivered twice daily in 4 fraction blocks repeated 4 weekly	Corry et al 2005^38^ (phase II, 35 patients)	Response rate, symptom control, quality of life, and toxicity. Overall and progression-free survival	53% objective response rate. Median overall survival 5.7 months (95% CI 3.4 to 9.3) and progression-free survival 3.1 months (2.2 to 6.1). 85% of patients experienced improved or stable dysphagia after treatment, 56% experienced improved pain control, 44% reported improved overall quality of life
**Bladder cancer**			
35 Gy in 10 fractions *v* 21 Gy in 3 fractions radiotherapy	Duchesne et al 2000^39^ (RCT, 500 patients (272 assessable at 3 months))	Symptomatic improvement at 3 months. Overall survival	No significant difference for any endpoint (overall survival HR 0.99 (0.82 to 1.21), P=0.933). 51.4% reported symptom improvement (P=0.421 for comparison between arms). In patients experiencing these symptoms initially; haematuria improved in 88%; urinary frequency in 82%; nocturia in 64%, and dysuria in 72% of assessable patients at 3 months after treatment. Median overall survival 7.5 months
**Rectal cancer**			
30-39 Gy in 10-13 fractions	Cameron et al 2016^40^ (prospective multicentre, 51 patients)	Symptomatic improvement at 3 months	Improvements in pain (77% (54% to 100%)), rectal dysfunction (90% (71% to 100%)), and bleeding (100%)
**Gynaecological malignancies**			
Any external radiotherapy or brachytherapy regimen delivered palliatively to the cervix	van Lonkhuijzen et al 2011^41^ (SR, 476 patients, 7 retrospective studies, 1 prospective study)	Symptomatic improvement	Wide heterogeneity in studies with variable time points and poor reporting limited this analysis. Bleeding improvement ranged from 45% to 100% of patients, pain reduction 31-100%, and discharge 15-100%. Toxicity not consistently reported
**Locally advanced prostate cancer**			
Any palliative radiotherapy regimen delivered to the prostate	Cameron et al 2014^42^ (SR, 315 patients, 9 retrospective studies)	Symptomatic improvement, quality of life, toxicity	Pooled response rates were 73% for haematuria, 80% pain, 63% bladder outlet obstruction, and 78% rectal symptoms. Toxicity was mild/moderate, though not systematically recorded. No reports of quality of life or patient reported outcomes

RCT=Randomised controlled trial, SR=Systematic review, all phase II studies were non-randomised. OR=odds ratio. HR=hazard ratio. QALY=quality adjusted life year. CNS=central nervous system. MRI=magnetic resonance imaging.

Given that radiotherapy can only ever address focal disease, these treatments should supplement, not replace holistic palliative care. Assessment and support for all physical, psychological, and social needs, with strong communication between services, are necessary. Palliative radiotherapy rarely improves overall survival, which is reported to be a median of 5.2 months in one observational study.^43^ For patients with particularly limited prognosis, careful consideration of the appropriate level of intervention is essential; the potential benefits of treatment may be outweighed by expected side effects and treatment burden.

### Pain due to bone metastases

Postmortem studies have detected bone metastases in up to 70% of patients with advanced cancer.^44^ Such metastases often cause localised pain and account for 35-40% of all palliative radiotherapy treatments.^45^ Pain may be constant or intermittent, can be neuropathic with a radiating dermatomal component and possible altered sensation, and often limits activities of daily living.^46^ Initial management combines analgesics and a holistic assessment of needs with interventions as required, such as home adaptations and walking aids.^47^ If, despite weak opioids, patients have persistent pain or side effects of medication, consider referral for radiotherapy.^48^ Metastases in long bones have a risk of pathological fracture. When this risk is assessed to be high, surgical stabilisation is often carried out before radiotherapy.^49^ ^50^ ^51^

Palliative radiotherapy provides pain relief in a median of 2-3 weeks for 60% of patients (table 1).^16^ ^17^ Where pain recurs, retreatment can be considered after at least four weeks to allow response.^52^ Intravenous bisphosphonates offered equivalent pain relief to single fraction radiotherapy for metastatic prostate cancer in a single randomised controlled trial.^19^ This may be an alternative option for patients with prostate cancer naïve to bisphosphonates.

### Symptoms due to locally advanced thoracic cancer

Lung cancer is the third commonest cancer in the UK and 28% of patients will present with locally advanced disease.^53^ ^54^ Thoracic symptoms include dyspnoea (50%), chest pain (28%), cough (40%), haemoptysis (10%), and dysphagia (7%).^22^ Some of these local symptoms can be successfully palliated in about two thirds of patients, although the success rate varies with symptoms. More information is provided in table 1.^20^ ^21^

Palliative radiotherapy to the mediastinum improved obstructive dysphagia from locally advanced oesophageal cancer in around two thirds of patients after a median of four weeks in a non-randomised phase I/II study.^25^ Given this delay in improvement and the risk of deterioration due to acute oesophagitis, patients with clinically significant dysphagia at baseline often undergo oesophageal stenting before radiotherapy.^55^ Radiotherapy improves durability of swallowing function after stenting.^26^ ^27^ However, for patients with very limited prognosis, stenting alone can provide rapid relief of dysphagia, and this group is unlikely to benefit from the addition of palliative radiotherapy.

Symptomatic radiation pneumonitis (occurring in <5%) can occur from six weeks to six months after treatment that includes the lungs.^20^ ^21^ Refer patients with cough and dyspnoea without another clear cause to the treating oncologist urgently for assessment and consideration of oral corticosteroids.

### Pain and neurological compromise due to malignant spinal cord compression

Malignant spinal cord compression occurs when vertebral disease compresses the cord, either directly or as a result of vertebral collapse. More rarely, intraspinal or epidural metastases occur. Back pain is common, often occurring before neurological signs and symptoms, including sensory and motor disturbance and loss of sphincter control. Symptom progression varies, from neurological deterioration over hours to a gradual decline over weeks. Urgent magnetic resonance imaging (MRI) is required to confirm the diagnosis, and oral dexamethasone 16 mg once daily (with proton pump inhibitor) is routinely administered.^56^ ^57^ Subsequent assessments target expected prognosis in order to guide management decisions.^58^ ^59^

The median overall survival after a diagnosis of malignant spinal cord compression is 3-4 months.^29^ ^60^ When predicted prognosis is more than six months, neurosurgical decompression may be considered before radiotherapy on the basis of a single randomised study showing improved neurological outcomes.^32^ Unfortunately, most patients have a prognosis of less than six months. For these patients, urgent palliative radiotherapy (within 24 hours of MRI confirmation) aims to reduce pain and retain or improve neurological function.^56^ The best neurological outcomes are seen in those retaining some movement before treatment or with gradual onset of neurological symptoms.^61^ For patients with established paraplegia, less than 10% regain mobility; in the absence of pain, and if the prognosis is very limited, holistic palliative care and appropriate social or nursing support may be more appropriate.^62^

Acute side effects reflect the vertebral level treated, while late radiation induced spinal cord myelopathy is rarely seen with palliative doses (<1%).^63^

### Symptoms due to brain metastases

Brain metastases occur in 20-40% of individuals with systemic cancer.^34^ Presentation can be with seizures, focal neurology, or symptoms of raised intracranial pressure (nausea, vomiting, and headaches). Prognostic indices help to tailor treatment to the individual patient.^64^ ^65^ For those with limited brain metastases and a life expectancy of more than six months, neurosurgery or stereotactic radiotherapy can be considered under discussion with the treating team, local neurosurgical or neuro-oncology teams, and patient.^36^ ^66^

For those with more extensive cerebral disease who retain a good performance status, whole brain radiotherapy can be offered, although no high quality randomised data exists to support this over corticosteroids alone.^67^ Indeed, a recent trial demonstrated no survival or quality of life benefit from whole brain radiotherapy over steroids alone in patients with brain metastases from non-small cell lung cancer.^33^ This has resulted in a reduction in the use of whole brain radiotherapy in this situation, but extrapolation to other cancer diagnoses is unlikely to be justified.

### Symptoms due to advanced head and neck cancer

Patients with locally advanced head and neck cancer often present with a range of difficult to control symptoms including pain, dysphagia or odynophagia, airway compromise, bleeding, and cosmetically distressing tumour bulk.^68^ These often frail patients have complex needs and require multidisciplinary support including specialist nursing and medical care, support from allied health professionals, palliative care, and community support with strong communication between services.

Prospective studies report improvement in pain control and quality of life in about 50-60% of patients after palliative radiotherapy, with improved ability to eat solids in 33%.^37^ ^38^ Of note, in one UK series, 18% of patients required hospital admission during or immediately after treatment for nutrition, dehydration, and pain control.^68^

### Symptoms due to advanced pelvic cancers

Locally advanced pelvic cancers can result in bleeding, discharge, bowel obstruction, urinary disturbance, and pelvic pain. Radiotherapy palliated bleeding in up to 90% of patients with advanced bladder, rectal, or gynaecological cancer and improved other symptoms for half to two thirds of patients.^39^ ^40^ ^42^ ^69^ Acute side effects frequently occur, alongside temporary deterioration of existing symptoms. If abdominal discomfort or diarrhoea are severe or fail to resolve with simple measures, seek advice from the treating oncology team.

### Bleeding, pain, and malodour due to skin cancers

Symptoms of bleeding, pain, and malodour due to advanced primary skin cancers responded to palliative radiotherapy in 61% of cases in a small observational study.^70^ Cutaneous disease—most commonly arising from breast cancer (metastases or primary), melanoma, and lung cancer^71^—can be treated similarly, although the evidence is extremely limited and there are no randomised comparisons with alternative approaches (such as surgical resection, electro-chemotherapy, photodynamic therapy, topical treatments).^72^ ^73^

## What are the most common side effects of palliative radiotherapy?

The side effects of radiotherapy are dictated by which tissues receive a substantial dose. For example, conventional radiotherapy to lumbar spine vertebral metastases will usually involve irradiation of the bowels, resulting in side effects related to both the bone metastasis and bowels (see fig 3). Additionally, treatment is associated with fatigue in at least two thirds of patients, and this can affect quality of life, limiting participation in preferred activities.^74^ ^75^

Acute side effects of palliative radiotherapy usually resolve within 4-6 weeks of completing treatment. In routine practice, palliative prescribing of analgesia (including strong opiates) and antiemetics underpins the management of side effects. Randomised evidence is limited, and the recommendations for management of side effects (see table 2) are predominantly based on systematic reviews and guidelines.

**Table 2:** Management of the acute side effects of palliative radiotherapy by organ or tissue

Anatomical site	Side effects	Management	Supporting evidence
Bone	35% of patient in the first week after treatment to bone metastases experience a pain flare. This resolves within a median of 3 days^76^ ^77^	Oral dexamethasone 8 mg once daily before treatment and for 4 days after, possibly with oral proton pump inhibitor	Rate of flare significantly reduced with dexamethasone (26% *v* 35%, P=0.05) (RCT, 298 patients)^76^
Lung	Cough after treatment is not well documented but common in practice	Routinely managed with medication (such as weak opioids)	Limited evidence supporting any specific intervention (SR, 326 patients, 9 studies)^78^
Mediastinum	Oesophagitis results in odynophagia or dysphagia in 14-22% of treated lung cancer patients (SR)^20^ ^21^ ^79^ and 28% of oesophageal cancer patients.^25^ Chest discomfort within the first few weeks after treatment	Antacid mixed with local anaesthetic, simple analgesia, proton pump inhibitors, and soft bland diet. Dietetic referral and enteral feeding maybe required, particularly in patients with compromised swallow before treatment	Recommendation based on a recent literature review as no randomised evidence was identified to inform acute supportive management^79^
Bowel or stomach	Nausea (such as seen during treatment to bone metastases in 61% or treatment for rectal cancer in 36%)^40^ ^80^	Antiemetics 30-60 minutes before, during, and after treatment (such as 5-HT_3_ receptor antagonists)	5-HT_3_ antagonists reduced emesis compared with conventional antiemetics or placebo (SR of RCTs) and are recommended in international guidelines^81^ ^82^
	Diarrhoea and abdominal discomfort during treatment for pelvic tumours in 20-40%,^39^ ^40^ ^42^ resolves within 6 weeks	Loperamide 2-4 mg and hyoscine butylbromide 20 mg as required. If diarrhoea severe (>6 bowel movements daily) or fails to improve within 12 hours, discuss with the treating oncology team	Recommendation based on regional guidelines and palliative prescribing as no randomised evidence identified^83^ ^84^
Bladder	Dysuria, frequency, and nocturia. During the first few weeks after treatment in 33% and 20% of bladder and prostate cancer patients treated to the primary tumour^39^ ^42^	Simple analgesia, good fluid intake, and anticholinergic agents are used in routine care. Cranberry capsules can be considered	Recommendation based on regional practise as no randomised evidence identified.^85^ Four small RCTs investigated role of cranberry supplements; two found reduced cystitis^86^
Brain	Fatigue	Exercise, as possible, has been shown to reduce fatigue in cancer patients generally	Standardised mean difference in fatigue −0.27 (95% CI −0.37 to −0.17) with exercise (MA, 2648 patients, 38 trials).^87^ Small RCTs of psycho-stimulants show mixed results in cancer related fatigue (SR).^88^-^90^ No evidence in whole brain radiotherapy specifically
	Headache (32%)^33^	Simple analgesia with dexamethasone 4 mg once daily if persistent	Recommendation based on routine palliative care prescribing^84^
	Nausea and vomiting (10-16%)	Antiemetics (such as cyclizine) and dexamethasone if persistent	Recommendation based on routine palliative care prescribing^84^
	Otitis externa (5%)	Otitis externa is often asymptomatic, steroid drops can be used if troublesome	No randomised evidence identified^91^
Skin	Sunburn-like erythema over treated area, peaks late in treatment and for about 10 days afterwards. Severity is dictated by dose	Daily washing, unperfumed emollient creams or soaps, and non-adhesive dressings. For more severe reactions (with skin breakdown) the treating department should be contacted for advice	Recommendation based on regional guidelines^92^ ^93^ as no strong conclusions reached in two literature reviews^94^-^96^
	Hair loss (most patients undergoing palliative radiotherapy to brain)^33^ ^97^ ^98^	Wig referral before treatment can be arranged, although timing this can be difficult in the palliative setting	Information and alternative approaches to hair loss are available through a variety of websites^99^ ^100^
Oral cavity and oropharynx	Oral or pharyngeal mucositis (63%) with pain and thickened secretions.^37^ Dysphagia (85%)^37^ and risk of aspiration pneumonia. Side effects peak at the end of treatment to two weeks beyond, then resolve over a month	Oral hygiene, regular mouth washes (such as saline, sodium bicarbonate), topical analgesia or gels, nebulised saline and analgesia (including NSAIDs and opiates in appropriate formulations). Concerns for swallowing safety and nutritional status should be discussed with the treating team	No strong conclusions were reached for the management of existing mucositis in multiple SRs.^101^-^103^ Recommendations reflect national and international guidelines.^101^ ^104^ ^105^ Enteral feeding was required in 12% of patients in one observational UK series^68^

RCT=randomised controlled trial, SR=systematic review, MA=meta-analysis, NSAID=non-steroidal anti-inflammatory drug.

Long term side effects are uncommon in palliative radiotherapy, and management of these is led by the treating team with multidisciplinary involvement when required.^106^

## What new treatments can we expect?

The radiotherapy dose delivered to a tumour is usually limited by likely side effects in surrounding tissues. Advanced techniques that offer treatments more closely matched to the tumour shape, delivered with computed tomography on the treatment couch immediately before radiotherapy, can more accurately target much higher radiotherapy doses to small focal disease sites. These more targeted stereotactic treatments are variously referred to as stereotactic body radiotherapy, stereotactic ablative body radiotherapy, and stereotactic radio-surgery. Figure 3 provides an example of the difference in radiotherapy dose distribution between the simple conventional palliative approach and these more complex treatments. 

There is now the potential for these higher dose stereotactic treatments to be used to improve survival and quality of life in patients with metastatic disease. This is being investigated for “oligo-metastatic disease” (in which a patient has only a limited number of metastatic deposits, and the disease has not become widespread).^115^ For such patients, high dose stereotactic treatments can be used to ablate all macroscopic sites of disease, potentially resulting in superior overall survival. However, even the existence of the oligo-metastatic state remains controversial.^110^ A further possible role for these treatments is in more advanced disease, where a higher radiotherapy dose to a symptomatic metastasis might provide better and more durable symptom control while continuing to deliver treatment in a minimum number of fractions with limited toxicity to surrounding tissues.^108^ ^109^ ^111^ There are no randomised data to support either of these approaches currently. Their expected value remains controversial, and trials are now under way for a range of indications.^107^ ^108^ ^109^ ^112^ ^113^ ^116^

An additional area of palliative radiotherapy in which significant advances are now being made is in the use of radionuclides. These treatments deliver radioactive isotopes to tumour tissue, either through anatomically targeted delivery (such as via the hepatic artery in metastatic colorectal cancer) or through the use of radiolabelled molecules or monoclonal antibodies which are preferentially taken up by the tumour or its microenvironment.^117^ Historically, their use has been limited to some relatively rare tumours, but novel agents are increasingly demonstrating benefits in a range of more common conditions such as metastatic prostate cancer.^114^ With trials ongoing internationally, these treatments are likely to be used more extensively over the next few years.

**A patient’s perspective**

My late husband received palliative radiotherapy multiple times during his treatment for multiple myeloma. Early in the course of his disease, radiotherapy for back pain and spinal cord compression ensured that he was able to continue the gardening he had always enjoyed. Receiving treatment was never uncomfortable for him, but, as his general condition deteriorated towards the end of his life, he spent more time in hospital and the benefits of radiotherapy became less clear. He had a mask made for one of his treatments which covered his head and neck: he didn’t find this particularly uncomfortable and he was excited to show it to everyone. He even let his grandchildren play with it once treatment was completed.

**Education into practice**

- Think about the last time you saw a patient with advanced cancer. How much did you consider localised disease as a possible cause of their symptoms?
- Would you feel confident referring them to discuss palliative radiotherapy to help treat their symptoms?
- What else might you do differently as a result of reading this article?

**How patients were involved in the creation of this article**

A patient representative (a relative of a previously treated patient) had the opportunity to review and comment on the draft manuscript. She did not feel any changes to the manuscript were needed but did share her experiences of her husband’s radiotherapy treatment.

## Notes

Provenance and peer review: Commissioned, based on an idea from the author; externally peer reviewed.
